# Correlation analysis between ApoM gene-promoter polymorphisms and coronary heart disease

**DOI:** 10.5830/CVJA-2016-001

**Published:** 2016

**Authors:** Yao Zhang, Li-Zhu Huang, Yan Liu, Qing-Ling Yang, Xin Zhou

**Affiliations:** Department of Biochemistry and Molecular Biology, Wan Nan Medical College, Anhui 241000, China; Clinical Testing and Diagnosis, Experimental Centre of Bengbu Medical College, Anhui 233000, China; Clinical Testing and Diagnosis, Experimental Centre of Bengbu Medical College, Anhui 233000, China; Department of Biochemistry and Molecular Biology, Beng Bu Medical College, Anhui 233000, China; Centre for Gene Diagnosis, Zhongnan Hospital, Wuhan University, Wuhan, Peoples’ Republic of China

**Keywords:** coronary heart disease, ApoM, SNP, luciferase activity

## Abstract

**Objectives:**

Apolipoprotein M (ApoM), a 25-kDa plasma protein belonging to the lipocalin protein family, is predominantly associated with high-density lipoprotein cholesterol (HDL-C). Studies have suggested ApoM to be important for the formation of pre-β-HDL and to increase cholesterol efflux from macrophage foam cells. The aim of this study was to explore the association of single-nucleotide polymorphisms(SNPs) in the ApoM promoter with coronary atherosclerotic disease (CAD), and the contribution of latent factors.

**Methods:**

ApoM was measured in samples from two separate case–control studies, of whom 88 patients developed CAD and 88 were controls. Whole-blood samples from subjects were genotyped by PCR-restriction fragment length polymorphism (PCR-RFLP). Luciferase activities were measured for HepG2 cells with two SNPs, rs805296 (T-778C) and rs940494 (T-855C), and after interfering with or overexpressing the predicted transcription factors. The ability of the SNPs to combine with nucleoproteins was analysed by electophoretic mobility shift assay (EMSA).

**Results:**

Mean plasma ApoM concentrations in the CAD and non-CAD groups were 9.58 ± 4.30 and 12.22 ± 6.59 μg/ ml, respectively. Correlation studies of ApoM concentrations with several analytes showed a marked positive correlation with HDL-C, fasting plasma glucose and triglyceride levels. The CC genotype showed lower luciferase activities compared to the TC and TT genotypes. The ApoM-855 mutant-typecould bind to the AP-2α. Interference and overexpression of AP-2 increased and decreased luciferase activities of the wild and mutant types to different degrees.

**Conclusion::**

ApoM may be a biomarker of CAD. ApoM- 855 T→C substitution provides binding sites for AP-2α and reduces ApoM transcription activity.

## Objectives

Apolipoprotein M (ApoM) is a novel lipocalin superfamily protein.^1^,^2^ Although also found in low-density lipoprotein (LDL), very low-density lipoprotein and chylomicrons, ApoM is primarily found in high-density lipoprotein (HDL) where it binds to sphingosine-1-phosphate (S1P) anchors.^3^

Recent studies have suggested that ApoM may affect HDL metabolism, increasing the formation of pre-β-HDL particles.^4^ ApoM has been shown to protect LDL against Cu^++^-induced oxidation,^5^ and to contribute to the anti-inflammatory function of HDL.^6^ Small circulating HDLs are involved in reverse cholesterol transport, and ApoM may affect this process by regulating pre-β-HDL.^7^ The binding of ApoM to S1P in HDL particles may also have an antioxidant role.

By affecting the immune and anti-inflammatory functions of HDL,^8^,^9^ ApoM may reduce atherosclerosis-related inflammation, preventing the onset and development of atherosclerosis. We used the online prediction software, TRANSFAC, to predict transcription factor (TF) binding sites for the normal and mutated ApoM-855 and ApoM-778 sites, and found ApoM T-855C provided binding sites for activating protein 2 (AP-2).

Activating protein-2α (AP-2α) was one of the first identified and studied TFs.^10^ The AP-2 gene, encoding a 437-amino acid protein of ~52 kDa, regulates the transcription of various genes regulating embryonic development, cell growth and differentiation.^11^,^12^ Vertebrates possess five subtypes of AP-2, α, β, γ, δ and ε.^13^,^14^

The AP-2 protein has been shown to regulate atherosclerosisassociated genes, including matrix metalloproteinase-2, vascular endothelial growth factor, ApoE, tryptase and adiponectin ATP-binding cassette transporter AI (ABCAI). In addition, AP-2α plays a role in atherosclerosis. It may mediate foam cell formation in mouse and human atherosclerotic lesions.^15^ AP-2α was found in ApoE^-/-^ mouse lesions within the artery wall, but was not detected in mouse arteries without atherosclerotic lesions. Similarly, AP-2α was observed in the human atherosclerotic aortic wall, mainly within the atherosclerotic plaque.

Recent studies involving the genetics of ApoM have led to major breakthroughs in metabolic and disease characteristics. In particular, associations have been found between diabetes and polymorphisms in the promoter region of the ApoM gene.^16^,^17^

To explore the association of ApoM gene polymorphisms with coronary heart disease (CHD) in a Chinese Han population, we performed a population-based case–control study. We examined whether ApoM promoter polymorphisms could lead to changes in TF binding, and therefore, changes in promoter activity.

## Methods

All relevant ethical and clinical approvals were obtained for this study. A total of 88 patients with coronary atherosclerotic disease (CAD) (63 males; mean age 60.80 ± 9.27 years) and 88 unrelated control individuals (53 males; mean age 58.18 ± 10.43 years) were retrospectively enrolled from among in-patients at the Anhui Cancer Hospital of Bengbu Medical College, Bengbu City, Anhui Province, China. All participants were of Han Chinese descent.

The criterion for inclusion in the CAD group was ≥ 50% stenosis in at least one major segment of the coronary arteries, determined by coronary artery angiography. Individuals in the control group had negative coronary artery angiography results (used to rule out CAD). A history of conventional risk factors for CAD or hypercholesterolaemia (total cholesterol ≥ 5.7 mmol/l) was obtained from the medical records. Exclusion criteria for both groups were familial hypercholesterolaemia, diabetes mellitus, cancer, renal disease and any other chronic illness.

For lipid analysis, whole blood samples were drawn from all participants in the morning after a 12-hour fast. Fasting plasma glucose (FPG), triglyceride (TG), HDL and LDL cholesterol (HDL-C and LDL-C), and total cholesterol (TC) levels were determined for each subject using an automated chemistry analyser (AU2000; Olympus Promarketing, Tokyo, Japan).

For ApoM analysis, 5-ml blood samples were collected in EDTA (as anticoagulant) after an overnight fast. Samples were centrifuged at 3 000 rpm for 10 minutes at room temperature. Separated sera were stored at –40°C. The serum ApoM level was quantified with ELISA, using horseradish peroxidase and the anti-ApoM antibodies 1E2 and 8F12 (Hunan Far Tai Biotechnology Co, Ltd). To minimise errors, all samples were used in the same reaction system, each test was repeated twice, and the average value was used in the final analysis.

## Genotyping of ApoM T-778C and T-855C polymorphisms

Genomic DNA was extracted from peripheral blood using a salting-out protocol. Single-nucleotide polymorphisms (SNPs) selected for this study are recorded in the public dbSNP database (http://www.ncbi.nlm.nih.gov/SNP/). Two SNPs, rs9404941 (T-855C) and rs805296 (T-778C), representing at least 5% of the minor allele frequency in the promoter region of the ApoM gene in the Han Chinese, were genotyped with the PCR-RFLP method. PCR primers for the ApoM T-855C and T-778C SNPs were designed based on GeneBank sequences Table 1.

**Table 1:** Primer, probe and oligonucleotide sequences used in this study

*Primer/probe/oligonucleotide*	*Sequence*
ApoM T-855C and T-778C SNPs	5′-GGTACCGTCTTTGCTAAGGGCTTTATGTGCATTA-3′ (forward)
	5′-AAGCTTTGTTGGTGTCAGGCAGAATGTGTCCAA-3′ (reverse)
Luciferase reporter plasmids	5′-AAGCTTCTCCTACTCGGGAATCAT-3′ (forward)
	5′ -GGTACCTCCAGAGCCTCCACCATA-3′ (reverse)
Wild-type T1 probe synthesis	5′ -TCGACATCCCAGGCTCAAGCAATCCT-3′
Wild-type T2 probe synthesis	5′-AGGATTGCTTGAGCCTGGGATGTCGA-3′
Mutant-type C1 probe synthesis	5′-TCGACATCCCAGGCCCAAGCAATCCT-3′
Mutant-type C2 probe synthesis	5′-AGGATTGCTTGGGCCTGGGATGTCGA-3′
	Sense 5′-UUCUCCGAACGUGUCACGUTT-3′
Negative control	Anti-sense 5′-ACGUGACACGUUCGGAGAATT-3′
Interference fragment 1	Sense 5′-CCAGAUCAAACUGUAAUUATT-3′
	Anti-sense 5′-UAAUUACAAGUUUGAUCUGGTT-3′
Interference fragment 2	Sense 5′-GGAAGAUCUUUAAGAGAAATT-3′
	Anti-sense 5′-UUUCUCUUAAAGAUCUUCCTT-3′
Interference fragment 3	Sense 5′-CCUGCUCAUCACUAGUATT-3′
	Anti-sense 5′-UACUAGUGAUGUGAGCAGGTT-3′
AP-2α forward primer	5′-CTGGGCACTGTAGGTCAATCT-3′
AP-2α reverse primer	5′-CCTCCTCGATGGCGTGAG-3′
GAPDH forward primer	5¢-CAAGGTCATCCATGACAACTTTG-3¢
GAPDH reverse primer	5¢-GTCCACCACCCTGTTGCTGTAG-3¢

PCR products were digested in a total volume of 20 μl containing 1 μg of PCR product, 5 U of restriction endonuclease HaeIII (-855) or RsaI (-778) (Fermentas, USA), 2 μg acetylated bovine serum albumin (BSA), and 2 μl restriction enzyme buffer. ApoM polymorphisms were detected with 2.5% agarose gel (Promega, USA) electrophoresis and visualised with ethidium bromide staining.

**Fig. 1.**
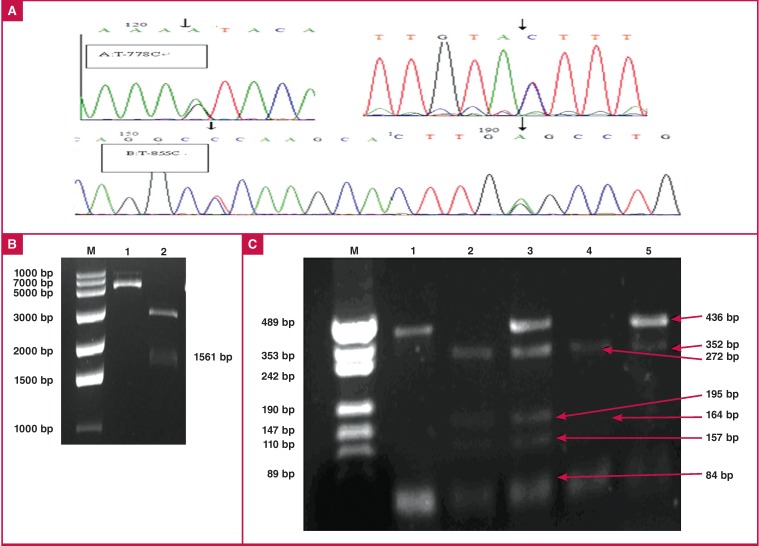
PCR-RFLP analysis results for the ApoM T-855/-778C locus. A. Sequencing map for PCR products of the two alleles of T-855C and T-778C. B. ApoM gene promoter pGL3 reconstruction vector (6379 bp) with restriction enzyme digestion. Lane M: ladder of molecular size markers; lane 1: vectors without restriction enzyme digestion; and lane 2: vectors with restriction enzyme digestion. C. Electrophoresis results of SNPs of ApoM proximal promoter and products separated on a 3% agarose gel and stained with ethidium bromide. Lane M: marker; lane 1: missed cleavages; lane 2: -T855C/C; lane 3: -T855C/TC; lane 4: T-778C/CC; lane 5: T-778C/TC.

In the T-778C polymorphism of the ApoM gene, the T→C substitution creates an RsaI restriction site. The PCR product with the C allele was digested into two fragments (272 and 164 bp), whereas the T allele was not cut by RsaI. Because T-855C contains GGC(C/T) and HaeIII recognises the GGCC sequence, digestion of TT homozygotes with HaeIII produced 496-, 352- and 84-bp fragments, CC homozygotes gave 84-, 157- and 195-bp products, and CT heterozygotes produced 84-, 157-, 195- and 436-bp fragments (Fig. 1C). To confirm detection of the T-778C polymorphism of the ApoM gene by PCR-RFLP, the PCR products were also sequenced (Shengong Bio Company, Shanghai, China; (Fig. 1C)).

## Construction of luciferase reporter plasmids

**Fig. 2.**
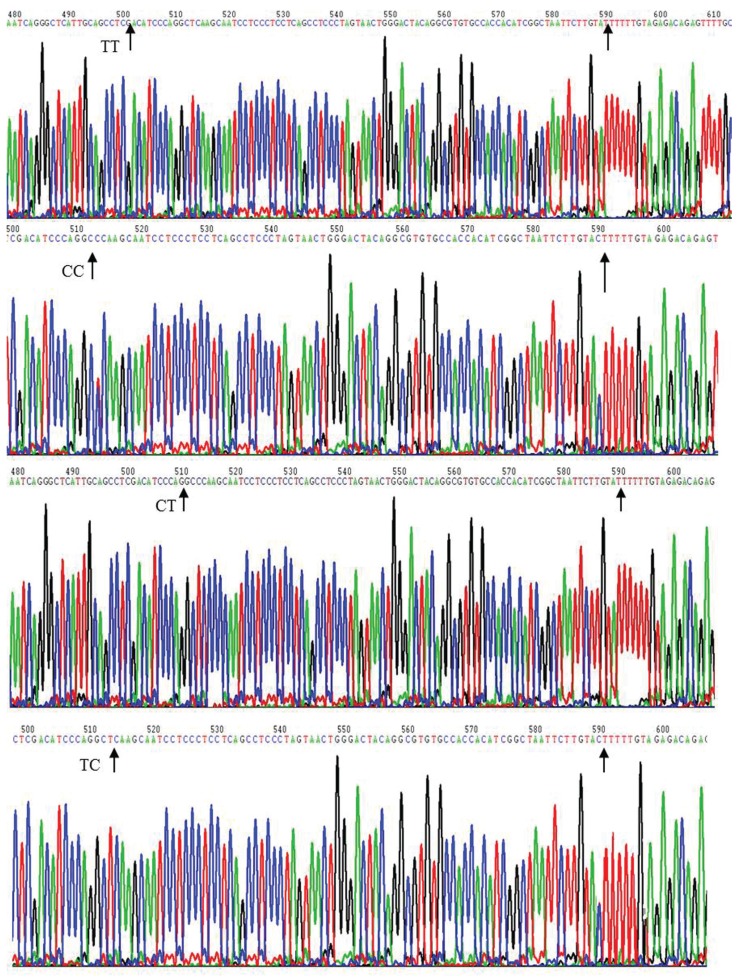
pGL3 recombination vector sequencing. PGL3-TT means the regions −855T to −778T; PGL3-TC means the regions −855T to –778C; PGL3-CT means the regions −855C to −778T; PGL3-CC means the regions −855C to −778C.

Four reporter plasmids, encompassing −1334 to +227 bp of the human ApoM promoter, were constructed. Plasmids containing the regions −855T to −778T, −855T to –778C, −855C to −778T and −855C to −778C were named PGL3-TT, PGL3-TC, PGL3- CT and PGL3-CC, respectively (Fig. 2).

PCR primers containing a KpnI- and HindIII-inserted forward and reverse primer, respectively, used in the construction of these plasmids, are shown in Table 1. PCR products were first ligated into the PMD®8-T vector (Takara, Dalian, China) and then subcloned into the pGL3-basic vector (Promega). All constructs used in this study were sequenced to confirm their authenticity (Fig. 2).

## Cell culture, transfection and reporter gene assays

The HepG2 hepatoma cell line was propagated in Dulbecco’s modified Eagle’s medium (DMEM; Hyclone) with 10% foetal calf serum (FCS) and maintained in 5% CO_2_ at 37°C. HepG2 cells were seeded in 24-well plates at 5 × 10^4^ HepG2 cells/ well, respectively. After 24 hours, the cells were transfected with pGL3-basic (a promoterless control), pGL3-promoter (a promoter control), and two pGL3-basic constructed plasmids. The pRL-SV40 plasmid (Promega) was co-transfected as a normalising control.

To prevent transcription of ApoM by endogenous liver cell extracts, recombinant vector transfection groups were set up with and without liver cell extracts. After six hours, the AP-2α siRNA and AP-2α siRNA negative control fragments (Gima) were co-transfected into the two pGL3-basic constructed plasmid groups. The AP-2α expression vector (GeneChem) groups were transfected into cells in the same way. Transfections were performed in triplicate and repeated three times. After 48 hours of incubation, the cells were collected and analysed for luciferase activity with the Dual-Luciferase Reporter assay system (Promega).

## Nuclear extract preparation

HepG2 cells were propagated in DMEM with 10% FCS under 5% CO_2_ at 37°C. Cells were cultured to a density of 1 × 10^7^ cells/ ml, according to the manufacturer’s instructions.

HepG2 cells were collected in a 15-ml centrifuge tube and centrifuged at 500 g for five minutes at 4°C. The supernatant was discarded, and the cells were washed three times with 2 ml of pre-cooled phosphate-buffered saline (PBS). For every 20 μl of cell sedimentation, 200 μl of reagent A were added. The mixture was vortexed for five seconds, which caused the cell sedimentation to disperse completely, and placed in an ice bath for 15 minutes. Then 10 μl of cell plasma protein extraction reagent B was added to the solution. The mixture was vortexed for five seconds, placed in an ice bath for one minute, vortexed again for five seconds, and centrifuged at 16 000 g for five minutes at 4°C.

The supernatant was removed and 50 μl of phenylmethylsulfonyl fluoride (PMSF) nuclear protein extraction reagent was added. The mixture was vortexed for 30 seconds, placed on ice for two minutes, and vortexed again for 30 seconds. This cycle was repeated for a total of 30 minutes. After a final centrifugation at 16 000 g for 10 minutes at 4°C, the nuclear extract was drawn into a pre-cooled 1.5-ml centrifuge tube.

## Electrophoretic mobility shift analysis (EMSA)

Sequences used for T1 and T1 wild-type and C1 and C2 mutanttype probe synthesis Table 1. were labelled and unlabelled with biotin, respectively. Nuclear cell extracts were incubated with biotin-labelled double-stranded (ds) oligonucleotide probes containing the wild-type or mutant AP-2α binding sites in the ApoM promoter (wild-type T1 and mutant-type C1 probes in Table 1.). Competition analysis was performed using the mutant probe with the AP-2α site. Supershifts were performed with antibodies against AP-2α and Sp1 (Abcam).

## Small interference (siRNA) transfection and efficiency

Three pairs of ds siRNA oligonucleotides were obtained from Gima Biotechnology Company, China Table 1.. Five groups were designated: a blank control group, a negative control group and three siRNA groups. Cells were seeded in six-well plates at 1 × 10^5^ cells/well, and then 1 μl of siRNA and 2 μl of siRNAMate were added to each well, corresponding to a density of 70 to 80% at the time of transfection. All of the steps were strictly performed according to the manufacturer’s specifications. Cells were harvested at 48 hours. The Sp1 siRNA and AP-2α expression vectors were transfected into cells in the same way.

Six hours after fluorescein amidite (FAM)-labelled siRNA transfection, fluorescence was observed under a fluorescence microscope. The transfection efficiency was determined as: efficiency = number of fluorescent cells/total number of cells × 100%.

## Semiquantitative RT-PCR

Total RNA was extracted from the cell clones using TRIzol reagent (Invitrogen). The cDNAs were reverse-transcribed from total RNA. The primers used are shown in Table 1.. The sizes of the PCR products for AP-2α and GAPDH were 385 bp and 496 bp, respectively. PCR products were checked by agarose gel electrophoresis. The abundance of each mRNA was detected and normalised to that of GAPDH mRNA.

## Western blotting analysis

Cells in all groups were collected after 72 hours, and the total protein was extracted with RIPA lysis buffer (Beyotime Institute of Biotechnology). The protein was measured with the BCA protein assay and diluted with cell lysate to an equal concentration in each group (40 μg protein/group). A 10% SDS-PAGE analysis was performed. The proteins were transferred to a PVDF membrane (250 mA, 2 h), blocked with 5% BSA in PBS containing Tween-20 (PBST), and incubated with a 1:500 dilution of anti-AP-2α overnight at 4°C.

The membrane was washed with TBST and incubated with a peroxidase-conjugated secondary antibody (1:1 000) for two hours. Specific antibody binding was detected using a chemiluminescence detection system, according to the manufacturer’s recommendations. Net intensities of the bands on the Western blots were quantified using Tanon GIS software. After development, the membrane was stripped and re-probed with antibody against β-actin (1:1 000) to confirm equal sample loading.

## Statistical analysis

Unless otherwise noted, results are reported as mean ± standard deviations (SD). General characteristics in the two groups and serum ApoM levels for different genotypes were statistically evaluated using the unpaired Student’s t-test (Prism software, version 4; GraphPad Inc, La Jolla, CA, USA). Significance was established at a p-value <0.05.

## Results

## Association between risk factors for CAD and ApoM levels in CAD patients

The general characteristics of age- and gender-matched non-CAD and CAD patients are shown in Table 2.. The mean ages of the non-CAD and CAD patients were 60.80 ± 9.27 and 58.18 ± 10.43, respectively (p > 0.05). The CAD patients had higher TG (1.97 ± 1.28 mmol/l) and FPG levels (6.40 ± 2.40 mmol/l), and lower HDL-C levels (1.05 ± 0.25 mmol/l) than the non-CAD patients (all p = 0.000).

**Table 2:** Clinical data for CAD and control groups

	CAD					
*Index*	*All CAD*	*ACS group*	*SAP group*	*p-value*	*Control*	*p-value*
Number (cases)	88	31	57		88	
Age (years)	60.80 ± 9.27	61.00 ± 8.68	62.96 ± 11.22	0.400	58.18 ± 10.43	0.081
Male/female	63/25	20/11	25/22	0.091	53/35	0.112
TC (mmol/l)	4.47 ± 1.41	5.17 ± 1.34	4.34 ± 1.44	0.010	4.33 ± 0.49	0.299
TG (mmol/l)	1.97 ± 1.28	2.20 ± 1.27	2.09 ± 1.32	0.698	1.02 ± 0.37	0.000#
HDL-C (mmol/l)	1.05 ± 0.25	1.05 ± 0.21	1.04 ± 0.27	0.951	1.32 ± 0.21	0.000#
LDL-C (mmol/l)	2.70 ± 1.23	3.01 ± 1.48	2.61 ± 1.05	0.140	2.53 ± 0.41	0.202
FPG (mmol/l)	6.40 ± 2.40	8.36 ± 4.11	11.58 ± 3.88	0.000*	4.86 ± 0.45	0.000#
ApoM (μg/ml)	9.58 ± 4.30	6.55 ± 2.74	6.32 ± 2.20	0.670	12.22 ± 6.59	0.037
Gensini score		80.48 ± 72.46	45.96 ± 51.00	0.017*		
1 branch	9	16				
2 branch	5	21				

Data are means ± SD. *Indicates statistical significance (p < 0.05) compared with ACS and SAP group.

#Indicates statistical significance (p < 0.05) compared to the control group with Dunnett’s test.

Because the Gensini scores were skewed distributions, Napierian logarithmic transformation was applied for normalisation.

CAD, coronary artery disease; ACS, acute coronary syndrome; SAP, stable angina pectoris; TC, total cholesterol; TG, triglyceride; HDL-C and LDL-C, high- and low-density lipoprotein cholesterol, respectively; FPG, fasting plasma glucose; ApoM, apolipoprotein M.

According to their clinical symptoms and the American College of Cardiology (ACC)/American Heart Association (AHA) diagnostic guidelines, patients in the CAD group were divided into two subgroups: acute coronary syndrome (ACS group, n = 31) and stable angina pectoris (SAP group, n = 57). The Gensini score was calculated for the CAD group and both CAD subgroups, according to CHD severity Table 2.. The ACS group had a higher average Gensini score (80.48 ± 72.46, p = 0.017), higher TC level, and lower ApoM level than the SAP group (all p > 0.05).

Analysis of the serum ApoM lipid levels of the patients showed that the serum level of ApoM was positively correlated with HDL-C and negatively correlated with LDL-C and TG levels (p < 0.05). The correlation with TC was not significant Table 3..

**Table 3:** Multiple linear regression analysis of serum ApoM

*Index*	*Partial regression coefficient*	*SD*	*Standard regression coefficient*	*t-value*	*p-value*
Constant	3.592	2.081		1.726	0.088
HDL-C	9.767	1.316	0.573	7.421	0.000*
FPG	–0.539	0.138	–0.301	–3.908	0.000*
TG	–0.464	0.217	–0.138	–2.136	0.036*

*Statistical significance (p < 0.05).

HDL-C, high-density lipoprotein cholesterol; FPG, fasting plasma glucose; TG, triglycerides.

We used the multiple linear stepwise regression method to describe the relationship between serum ApoM and HDL-C and LDL-C levels. Linear dependencies and other related indicators were used as dependent variables (ŷ). ApoM was used as the independent variable (x). When HDL-C, FPG and TG were used as three indicators in the regression equation (x_1_, x_2_ and x_3_, respectively), the resulting general multiple regression equation was ŷ = 3.592 + 9.767x_1_ – 0.539x_2_ – 0.464x_3_ with the regression coefficient test, with p = 0.000 and a coefficient of determination of 0.651. The standardised regression equation was ŷ = 0.573x_1_ – 0.301x_2_ – 0.138x_3_. The closest relationship was between serum HDL-C and ApoM levels Table 3..

## Association of polymorphisms in the ApoM proximal promoter region with CAD

DNA sequencing of the polymorphic regions in the proximal promoter of the ApoM gene revealed that SNPs T-778C and T-855C of the ApoM gene were valid in the Han Chinese. The SNPs were accurately detected by PCR-RFLP (Fig. 1). Table 4. shows the plasma lipid and ApoM levels in each group according to the SNP status.

**Table 4:** Lipid profiles according to genotype

	CAD			Control		
*Lipid parameter*	*TT*	*CT+CC*	*p-value*	*TT*	*CT+CC*	*p-value*
rs805296						
TC (mmol/l)	4.61 ± 1.37	4.77 ± 1.86	0.030#	4.33 ± 0.49	4.31 ± 0.52	0.900
TG (mmol/l)	1.93 ± 1.01	3.05 ± 2.02	0.002#	1.03 ± 0.82	0.82 ± 0.32	0.157
LDL-C (mmol/l)	2.78 ± 1.18	2.60 ± 1.46	0.610	2.53 ± 0.41	2.60 ± 0.44	0.655
HDL-C (mmol/l)	1.05 ± 0.23	1.01 ± 0.34	0.557	1.32 ± 0.22	1.35 ± 0.19	0.706
ApoM (μg/ml)	10.35 ± 4.41	6.46 ± 4.06	0.009#	13.22 ± 9.18	3.57 ± 3.86	0.007#
Rs9404941						
TC (mmol/l)	4.43 ± 1.39	5.30 ± 1.49	0.017#	4.34 ± 0.49	4.23 ± 0.54	0.542
TG (mmol/l)	1.95 ± 1.32	2.70 ± 1.04	0.020#	1.02 ± 0.38	0.97 ± 0.24	0.711
LDL-C (mmol/l)	2.66 ± 1.11	3.03 ± 1.52	0.237	2.54 ± 0.40	2.46 ± 0.50	0.604
HDL-C (mmol/l)	1.07 ± 0.26	0.97 ± 0.20	0.123	1.32 ± 0.21	1.32 ± 0.26	0.990
ApoM (μg/ml)	10.17 ± 5.68	6.07 ± 4.70	0.009#	12.87 ± 9.40	8.30 ± 6.45	0.184

Data are means ± SD. #Statistical significance (p <0.05) for TC/CC genotype group vs TT group with Dunnett’s test.

TC, total cholesterol; TG, triglyceride; LDL-C, HDL-C, low- and high-density lipoprotein cholesterol, respectively; ApoM, apolipoprotein M.

In the non-CAD group, we found no significant difference in lipid plasma levels between the TT and the TC+CC groups. In the CAD group, the TC, TG and ApoM levels were significantly different between the TT and TC/CC groups. The ApoM plasma levels in the TT and TC+CC groups were 10.35 ± 4.41 and 6.46 ± 4.06 μg/ml, respectively in T-778C, and 10.17 ± 5.68 and 6.07 ± 4.70 μg/ml, respectively in T-855C (both p < 0.05; Table 4.).

## PCR amplification of the human ApoM gene promoter region

From the above results, that patients with the TC/CC genotype showed lower plasma ApoM levels compared to those with the TT genotype, and that ApoM levels were lower in CAD compared to non-CAD patients, we inferred that the ApoM promoter variation may alter the promoter activity. To verify whether the −778T→C and −855T→C variation affected the ApoM promoter activity, we applied a reporter gene assay to detect the luciferase expression with transfection of the specific allele(s) of the polymorphism.

**Fig. 3.**
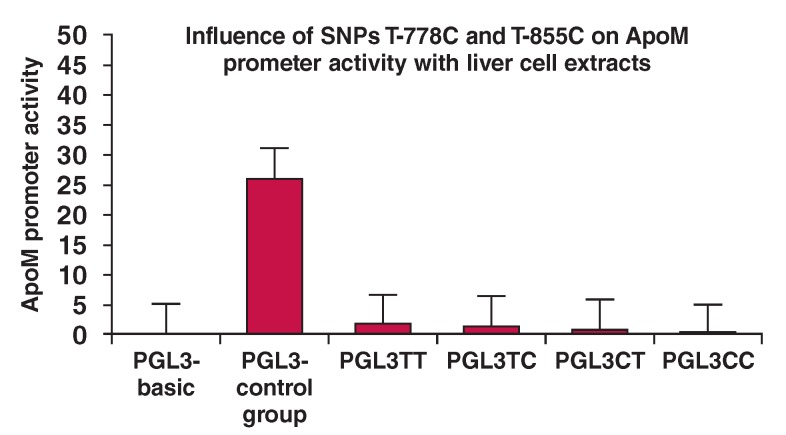
Relative luciferase activity of the ApoM promoter for each transfected group. Data are means ± SD. All groups were significantly different (p <0.05 by Dunnett’s test) compared to the PGL3-TT group.

We designed PCR primers to amplify a 1562-bp slice of the promoter region of ApoM. Four reporter plasmids, containing regions −855T to −778T, −855T to –778C, −855C to −778T and −855C to −778C, were named PGL3-TT, PGL3-TC, PGL3- CT and PGL3-CC, respectively. The luciferase activities of the PGL3-TC/CC promoters were lower than those of the PGL3-TT promoter (PGL-TT, 1.67 ± 0.14; PGL-TC, 1.28 ± 0.11; PGL-TC, 0.77 ± 0.21; PGL-CC, 0.25 ± 0.10, p = 0.001; (Fig. 3))

## Transcription factor prediction

**Fig. 4.**
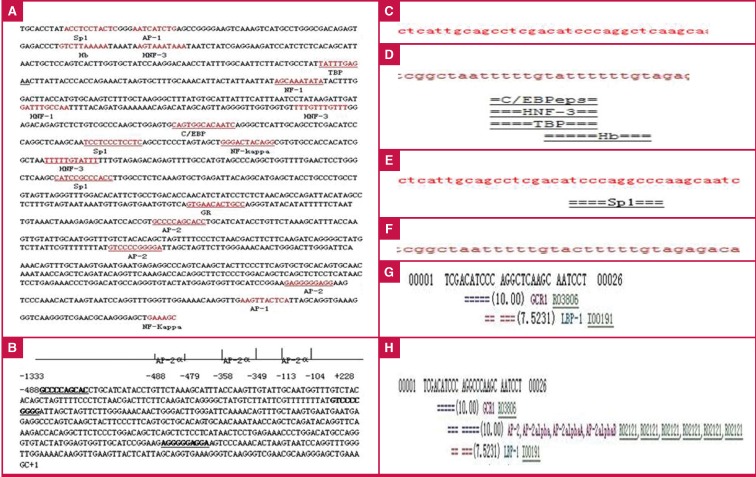
Prediction of transcription factor binding in the ApoM promoter region. A. Transcription factor binding prediction for ApoM. B. AP-2α potential binding sites of ApoM start promoter sequence. Mutation site analysis for ApoM-855 and ApoM-778 by TRANSFAC (C–F), and by TESS (G–H). Binding sites with wild-type alleles of ApoM-855 (C), and ApoM-778 (D), and binding sites with mutant-type alleles of ApoM-855 (E), and ApoM-778 (F) by TRANSFAC. Binding sites with wild-type (C), and mutant-type alleles (D) of ApoM-855 by TESS.

The TRANSFAC online prediction software was used to predict the combined TF binding sites upstream of the ApoM gene (Fig. 4A, B).. The binding sites were identified on the ApoM promoter for some TFs, including Sp1 and AP-1. The AP-2 binding sites were located at -479 to -488 bp, -349 to -358 bp and -104 to -113 bp on the ApoM promoter. TRANSFAC was used to predict TF binding sites for the normal and mutated ApoM-855 and ApoM- 778 sites (Fig. 4C-F).

ApoM T-855C provided binding sites for AP-2. ApoM-778T but not ApoM-778C had binding sites for hepatocyte nuclear factor-3 (HNF-3), CCAAT enhancer binding protein (C/EBP) and TATA box-binding protein (TBP). We also used the TESS online software to predict TF binding sites of the normal and mutated ApoM-855 (Fig. 4G, H). The results showed that ApoM T-855C provided binding sites for AP-2.

## EMSA results

Small molecular weight chains have faster mobility in EMSA, whereas the electrophoretic velocity will vary with probe and protein binding. The velocity of the electrophoretic band indicates the presence or absence of binding.

**Fig. 5.**
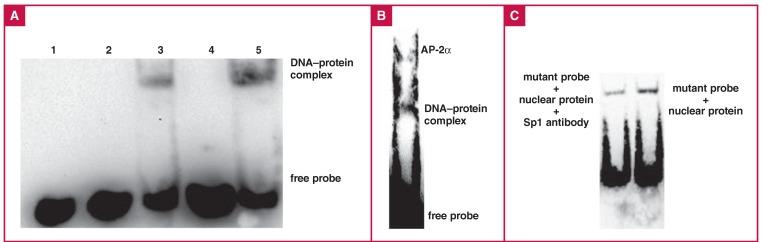
Experimental results for transcription factor binding in the ApoM promoter region. A. EMSA. Lane 1: biotin-labelled T probe + nuclear protein (NE); lane 2: biotin-labelled C probe; lane 3: biotin-labelled C probe + NE; lane 4: biotin-labelled C probe + NE + unlabelled C probe; and lane 5: biotin-labelled C probe + NE + unlabelled T probe. B. AP-2α supershift. Biotin-labelled C probe + NE + AP-2α antibody. C. Sp1 supershift. Biotin-labelled C probe + NE + Sp1 antibody.

We observed a hysteresis band when the C probe but not the T probe was incubated with nuclear protein (Fig. 5A). The competitive inhibition test showed that the hysteresis band of the C probe incubated with nuclear protein could be suppressed by unlabelled C probe, but not by unlabelled T probe (Fig. 5A).

**Fig. 6.**
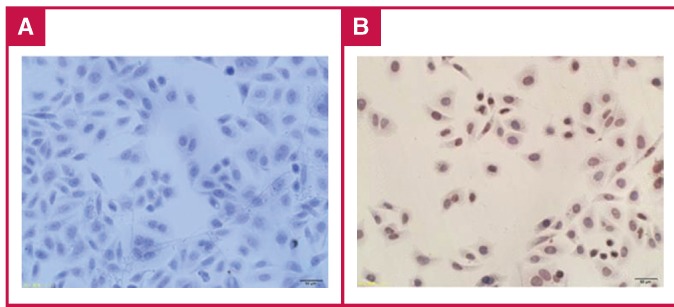
Confirmation of EMSA supershift results. To determine whether unbound Sp1 existed with the Sp1 antibody, we conducted cell immunohistochemistry experiments. Cells expressing Sp1 were dyed brown–yellow (B), and negative control cells were stained blue (A). The results indicated that the extracted nucleoprotein contained Sp1, and there was no problem in terms of the Sp1 antibody quality. These results further confirmed the EMSA supershift results.

The supershift test showed that incubating the C probe with the AP-2α antibody produced a more lagging band (Fig. 5B)., whereas the Sp1 antibody did not have this effect (Fig. 5C). The antibody activity of Sp1 was verified by immunohistochemistry analyses (Fig. 6A).

## Luciferase activity detection with AP-2α overexpression or interference

**Fig. 7.**
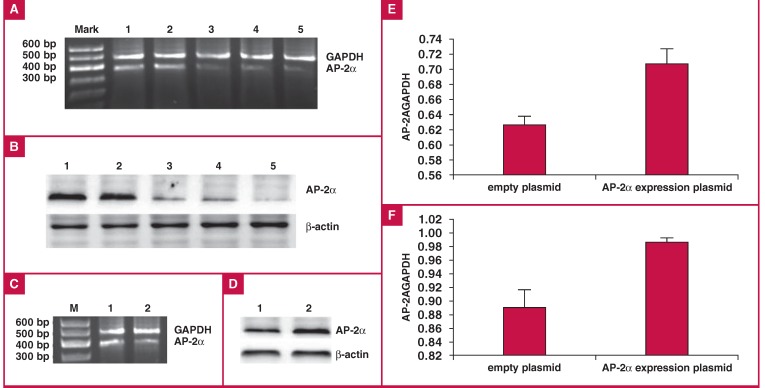
Efficiency of interference in siRNA fragments. After we determined the transfection efficiency of the fluorescent cells to be > 70%, we transfected HepG2 cells with siRNAs against AP-2α fragments and tested the ability of these fragments to interfere with AP-2α mRNA and protein production (A, B). Interference efficiency was calculated using the Tanon GIS image analysis system. The interference efficiencies of fragments 1, 2, and 3 on the AP-2α mRNA levels were 86.06, 83.68 and 92.43%, respectively, and on the AP-2α protein levels were 61.15, 58.42 and 79.59%, respectively. Because the interference efficiency of fragment 3 was the highest, we selected this fragment for use in the subsequent experiments. Interference and overexpression of AP-2α in HepG2 cells. RT-PCR (A) and Western blot analyses (B) of AP-2α mRNA and protein expression levels, respectively, in HepG2 cells treated with three interference fragments for 72 hours. AP-2α overexpression in HepG2 cells resulted in elevated AP-2α RNA (C), and protein expression levels (D), compared to controls. AP-2α mRNA vs GAPDH mRNA: 0.63 ± 0.01 vs 0.71 ± 0.02 (E); AP-2α protein vs β-actin protein: 0.89 ± 0.03 vs 0.99 ± 0.01 (F), both p < 0.05 by paired t-test.

**Fig. 8.**
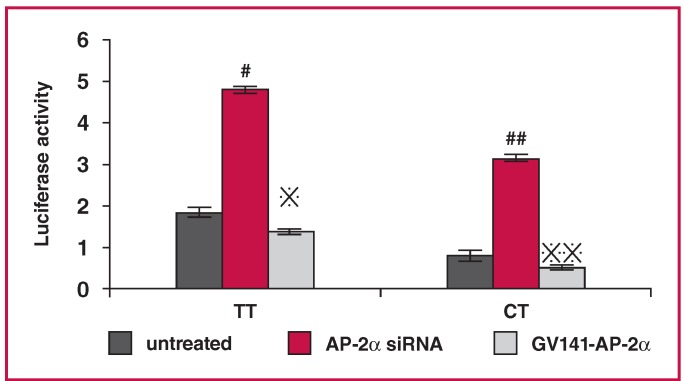
Relative luciferase activity of the ApoM promoter in each transfected group. For TT groups: untreated, 1.84 ± 0.12; TT + AP-2α, 4.83 ± 0.08; TT + GV141-AP-2α, 1.38 ± 0.06; For CT groups: untreated, 0.80 ± 0.08; CT + AP-2α, 3.19 ± 0.07; CT + GV141-AP-2α, 0.51 ± 0.05; PGL3-basic was used as a negative control and PGL3-control as a positive control. #p < 0.05 and p < 0.05 vs -855 wild-type untreated group and ##p < 0.05 and p < 0.05 vs -855 mutant untreated group by paired t-test.

To examine the role of the TF AP-2α in mediating ApoM promoter activity, we treated HepG2 cells expressing the mutanttype or wild-type allele of the ApoM-855 site with siRNAs against AP-2α (Fig. 7A, B) or with an AP-2α expression vector (Fig. 7C-F), and examined changes in the luciferase activity of the -855 site (Fig. 8).

With AP-2α interference, the luciferase activity in cells expressing the wild-type allele (1.63) was less elevated compared to cells expressing the mutant-type allele (2.99). The luciferase activities of treated cells were increased compared to the untreated control cells (p < 0.05).

With AP-2α overexpression, the activity levels of cells expressing the wild-type allele were more reduced than those of cells expressing the mutant-type (0.25 vs 0.38). The luciferase activity levels of AP-2α overexpressing cells were lower than those of the untreated cells (p < 0.05). Taken together, these results suggest that AP-2α was a negative regulatory factor for ApoM expression.

## Discussion

The results of this study confirm the previously identified^18^,^19^ association between CHD and SNPs T-855C and T-778C inthe promoter region of the ApoM gene. Luciferase activity associated with the -855 T→C substitution was significantly less than that of the promoter with -855 TT. With software predictive analysis we found the possible reason for this finding was that the -855 T→C substitution permitted the TFs AP-2α and Sp1 to bind to the promoter.

When HepG2 cells were transfected with the ApoM promoter containing the -855 T→C substitution, AP-2α combined with the ApoM-855 area, thereby decreasing promoter activity. These findings confirm that changes in the activation of the ApoM promoter region may induce variations in the ApoM plasma concentration.

Our results suggest that C alleles at the ApoM promoter -855 and -778 were associated with increased CHD risk. In our population-based case–control study, we enrolled 88 CHD patients (63 males, mean age: 60.80 ± 9.27 years) and 88 unrelated individuals (53 males, mean age: 58.18 ± 10.43 years) as a control group. The CHD group was divided into ACS and SAP groups, and the plasma levels of TG, TC, HDL-C, FPG and LDL-C were evaluated. Genomic DNA from whole blood of these subjects was subjected to PCR amplification and restriction enzyme digestion to determine genotype with regard to the ApoM T-855C and T-778C polymorphisms.

CHD patients had higher TG (1.97 ± 1.28 mmol/l; p = 0.000) and FPG levels (6.40 ± 2.40 mmol/l; p = 0.000), and lower HDL-C levels (1.05 ± 0.25 mmol/l; p = 0.000) than non-CHD patients. The allelic frequencies were in Hardy–Weinberg equilibrium.

After adjustment for age, gender and serum glucose level, multiple logistic regression analysis showed that, compared to the wild-type TT genotype of the two SNPs, carriers of the C allele had an increased risk of CHD, with an odds ratio (OR) of 1.819, 95% confidence interval (CI) of 1.142–2.898, and p = 0.012 (T-855C: OR = 3.206, 95% CI = 1.139–2.204, p = 0.037; T-778C: OR = 3.290, 95% CI = 1.487–7.280, p = 0.004). Luciferase activities of the promoter constructs with CC were significantly lower than those of the constructs with TC and TT

To detect whether different alleles of the ApoM proximal promoter region may affect the expression of target genes, thereby affecting the metabolism of ApoM, we constructed different genotypes of the promoter reporter gene to examine how mutations in the ApoM proximal promoter would affect promoter activity. The presence of a C allele at -855 or -778 bp of the ApoM promoter region may lead to lower ApoM levels and allow prediction of disease severity in the patient. However, due to the small number of cases analysed, more clinical data are needed for this conclusion to be confirmed.

We investigated whether the DNA sequence of ApoM from -844 to -869 bp was involved in transcriptional regulation of the ApoM gene. Using EMSA experiments, we showed that the mutant allele (-855C) could bind with nuclear proteins, whereas the wild-type allele (-855T) could not. Competitive inhibition experiments showed that the combination was due to specific binding by the TF AP-2α.

To explore the role of AP-2α in ApoM promoter activity, we examined the luciferase activities of the wild-type and mutant-type alleles after interference of AP-2α. Whereas AP-2α interference increased the luciferase activities of the treated cells, the wild-type was elevated to a lesser extent than the mutant-type. There were other AP-2α binding sites in addition to the -855 site.

These results suggest that AP-2α may be a negative regulatory factor of ApoM. The increased luciferase activity of the mutant type with Apo-2α interference compared to the wild-type may indicate that the mutant had more binding sites for AP-2α, or that the mutated -855 site can bind with AP-2α.

Multiple epidemiological studies have shown that serum HDL levels are negatively correlated with the risk of early CHD.^20^ Generally, clinical CAD is divided into two major types, ACS and SAP. Patients with ACS had significantly lower ApoM levels, probably due to the fact that ApoM is a major apolipoprotein of HDL.

It has been confirmed that ApoM is required for pre-β- HDL formation and cholesterol efflux to HDL, and that it protects against atherosclerosis.^4^ ApoM increased formation ofpre-β-HDL particles and had a profoundly protective effect on atherosclerotic lesion formation in hypercholesterolaemic Ldlr^-/-^ mice.^4^ Atherosclerotic lesion areas in aortic roots and the thoracic aorta were reduced in Ldlr^-/-^ mice infected with Ad-ApoM.^4^

The unstable lesion (also vulnerable plaque, the formation being mainly due to dyslipidaemia) is the basic pathological aetiological factor of ACS. Therefore the presence of an enlarged unstable lesion may be because the decreased serum ApoM level prohibited the formation of sufficient amounts of mature, functional HDL to promote the mobilisation of cellular cholesterol in vivo.^21^

The serum glucose level was different between CHD patients and normal controls, and serum ApoM and serum glucose levels were negatively correlated (both p <0.05). Very low ApoM levels increase the risk of atherosclerosis.^22^ Therefore the serum ApoMlevel may be a valuable marker for identifying high-risk groups.

TFs are the most important regulators of protein expression by genes and the most important factors to influence ApoM expression. Gene promoter regulation may underlie the low ApoM levels in CHD patients. Recent studies have shown that several TFs participate in the regulation of ApoM expression, such as HNF-1α, liver receptor homolog-1 (LRH-1), forkhead box A2 (Foxa2),^23^,^24^ liver X-activated receptor (LXR),^25^,^26^ leptin,^27^ interleukin-1 (IL-1),^28^ transforming growth factor (TGF) and epidermal growth factor (EGF).^9^

Our study has some limitations. Although our results confirm findings on the effect of the T-778C polymorphism on CHD, our analysis of the association of the ApoM promoter region SNPs with CHD was limited to a single locus. Such single-locus associations may be different in different populations. We found that the ApoM plasma concentration was decreased in CHD patients, the rs805296 and rs9404941 SNPs were associated with CHD occurrence and severity, and the rs9404941 SNP was associated with plasma TC and TG changes. However, the small sample size of this study limits its statistical power, and the results should be replicated in studies with larger sample sizes to avoid false positives. Expression of the ApoM protein and its relationship with diseases need to be further studied and discussed.

## Conclusion

ApoM may be a biomarker of CAD. ApoM-855 T→C substitution provides binding sites for AP-2α and reduces ApoM transcription activity.
